# Supplementary material for: SillyPutty: Improved clustering by optimizing the silhouette width
Source: PLoS One. 2024 Jun 7;19(6):e0300358. doi: 10.1371/journal.pone.0300358 (PMC11161052; doi:10.1371/journal.pone.0300358)
Supplement: S1 Fig — (PDF) [file pone.0300358.s001.pdf]

SillyPutty: Improved clustering by optimizing the silhouette width

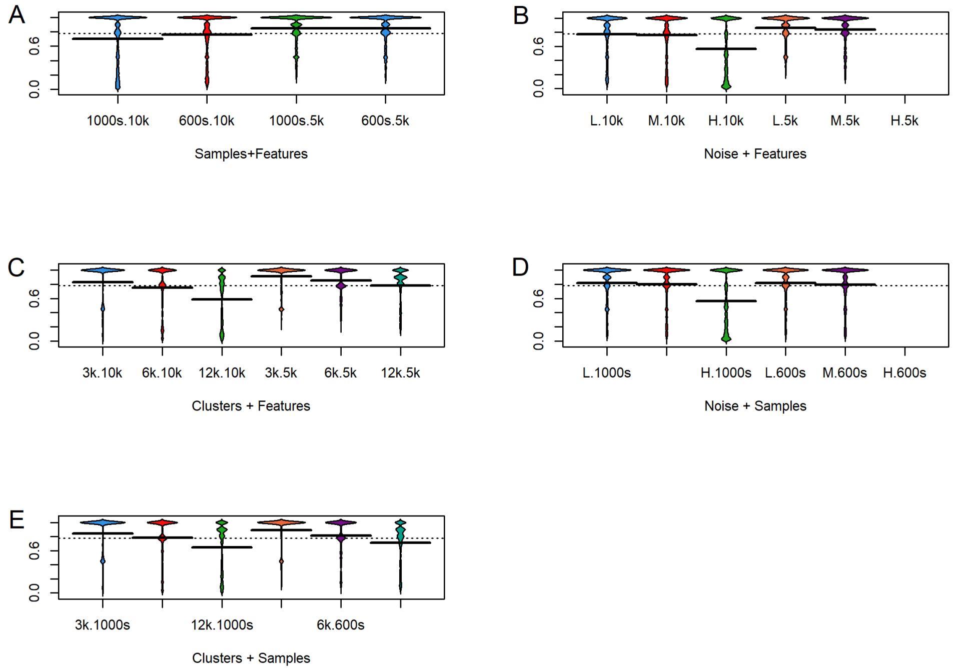

Figure 1: Effect of different combinations of parameters on ARI. (A) Samples and Features. (B) Noise and Features. (C) Clusters and Features. (D) Noise and Samples. (E) CLusters and Samples
